# Comparative Hair Restorer Efficacy of Medicinal Herb on Nude (Foxn1^nu^) Mice

**DOI:** 10.1155/2014/319795

**Published:** 2014-11-13

**Authors:** Shahnaz Begum, Mi Ra Lee, Li Juan Gu, Md. Jamil Hossain, Hyun Kyoung Kim, Chang Keun Sung

**Affiliations:** ^1^Department of Food Science and Technology, College of Agriculture and Biotechnology, Chungnam National University, Daejeon 305-764, Republic of Korea; ^2^Molecular Genetics and Genomics Lab, Department of Horticulture, Chungnam National University, Daejeon 305-764, Republic of Korea

## Abstract

*Eclipta alba* (L.) Hassk, *Asiasarum sieboldii* (Miq.) F. Maek (*Asiasari radix*), and *Panax ginseng* C. A. Mey (red ginseng) are traditionally acclaimed for therapeutic properties of various human ailments. Synergistic effect of each standardized plant extract was investigated for hair growth potential on nude mice, as these mutant mice genetically lack hair due to abnormal keratinization. Dried plant samples were ground and extracted by methanol. Topical application was performed on the back of nude mice daily up to completion of two hair growth generations. The hair density and length of *Eclipta alba* treated mice were increased significantly (*P* > 0.001) than control mice. Hair growth area was also distinctly visible in *Eclipta alba* treated mice. On the other hand, *Asiasari radix* and *Panax ginseng* treated mice developing hair loss were recognized from the abortive boundaries of hair coverage. Histomorphometric observation of nude mice skin samples revealed an increase in number of hair follicles (HFs). The presence of follicular keratinocytes was confirmed by BrdU labeling, S-phase cells in HFs. Therefore, *Eclipta alba* extract and/or phytochemicals strongly displayed incomparability of hair growth promotion activity than others. Thus, the standardized *Eclipta alba* extract can be used as an effective, alternative, and complementary treatment against hair loss.

## 1. Introduction

Hair is a dynamic mini-organ of mammalian skin that originates from the hair follicle and serves multiple functions throughout adult life. Inside the skin every HF is particularly active and is composed of fully keratinized epithelial cells. Hair growth is coordinated by hormones and it commands the follicle to undergo appropriate changes during this process. This process is followed by specific cyclic order and characterized by anagen (growth phase), catagen (regression), and telogen (resting phase) [1]. Human hair follicles also pose a unique paradox as the hormones, androgens, growth factor may cause stimulation of hair growth. On the other hand, genetically acquired disorder inhibit to generate well differentiated hair follicles or shorten anagen phase; these phenomena may cause baldness pattern [2, 3]. Although human hair loss is not life threatening, alopecia (hair loss/balding) may cause serious psychological distress [4]. These may reflect on some sociopsychological contexts. Novel therapies are therefore required to prevent hair loss and promote hair growth. In this context, developing alternative medicine has increased interest in the recent years. Until now, complete lack of strong scientific evidence of effective component of natural plant extract and limited to their hair promotion mechanisms has kept natural hair care away from mainstream research. Although the search for the treatment hair loss research resulted in some synthetic drugs, associated side effects are highly remarkable. Therefore, herbal products now emerged as an alternate to the synthetic drugs in this current state.

Studies of various research groups reported that hundreds of plants or substances are having potential to promote hair growth as well. In particular only little candidate plant plays great role to enhance hair growth whose efficacies are now under investigation. Therefore, recent research was under taken to investigate of some known plants those were traditionally acclaimed and purported in oriental medicine such as* A. radix *[5],* P. ginseng *[6, 7], and* E. alba *[8, 9] exert to promote hair growth.

Moreover,* E. alba* has also been reported with multiple medicinal uses and is known for numerous biological activities. It is an enormous source of several secondary metabolites, such as polypeptides, polyacetylenes and triterpenes [10], flavonoids, phytosterols, and coumestans [11]. Phytochemical coumestans represent an important class of natural oxygenated aromatic compound, including wedelolactone and demethylwedelolactone, and saponins, responsible for the main medicinal effects of* E. alba*, such as its antihepatitis C virus [12], antitumor activity [13], and neuropharmacological activity [14] and its anticancer effect shows antiproliferative and cytotoxic effects determined using MTT assay [15].

Taken together this bioresource information, we undertake nude mice as a model to get more precise result for hair biology from different well-known medicinal plant extracts, as these mice express the role of Foxn1 in mammalian skin biology responsible for structural abnormality of hair shaft due to abnormal keratinization [16]. Moreover, nude mice are also frequently used in skin pharmacology to serve for skin penetration assays [17] as the follicular penetration route acts as an entry pathway for compounds into the skin [18].

*E. alba* is widely reported traditional medicine and wellbeing functional food, thus it prompted us to evaluate hair growth promoting activity along with other plants. Therefore, this approach may incorporate some novel findings that may promote us to explore extensive bioactive compounds from crude extract for prevention of hair loss as well as enhancing hair growth.

## 2. Materials and Methods

### 2.1. Plant Materials and Preparation of Extraction

Aerial parts (leaves, stems, and blooms) of* E. alba* and dried roots of* A. radix* and* P. ginseng* were purchased from the Jecheon Medicinal Herb Association, South Korea. All the samples were authenticated by Associate Professor Ki Hwan Bae (College of Pharmacy, Chungnam National University) where the voucher specimens were deposited. The dried plant sampleswere ground into powder (500 g) and extracted with 95% methanol at 60°C. The methanol extract was concentrated and dried in a vacuum evaporator under reduced pressure below 60°C. The resulting residue was weighed and dissolved in a vehicle mixture (propylene glycol : ethanol : dimethyl sulfoxide, 67 : 30 : 3% v/v). The extract yields were determined as “%” of dry crude extracts (Table 1).

### 2.2. Chromatographic Analysis

HPLC-grade acetonitrile was purchased from Merck Co. (Merck, Darmstadt, Germany). Deionized water was purified by Milli-Q system (Millipore, Bedford, MA, USA). Other chemicals were of reagent grade. Standard wedelolactone powder was purchased from the Sigma-Aldrich Co. The purity of the standard was over 98% as indicated by the manufacturer. Calibration standard samples were prepared from the stock solutions by adding 0.5–1.5 mg/mL of wedelolactone in MeOH to obtain appropriated dilutions which were filtered through Millipore membrane before use. Chromatographic analysis was performed on the Shimadzu (Japan) HPLC apparatus, with a column of Discovery C18 (ODS, 25 cm × 4.6 mm, 5 *μ*m, Supelco, USA) and a Guard Column: Security Guard Cartridge system (Phenomenex, USA). The mobile phase consisted of acetonitrile: water (ACN%, v/v) at a flow rate of 1 mL/min as follows: 20–45% (up to 15 min), 45–100% (up to 30 min), and 100% (up to 40 min). The method was performed with an injection volume of 20 *μ*L and detection wavelength at 365 nm.

### 2.3. Toxicity Studies

Toxicity studies were carried out by applying the extract to the nude mice skin in concentrations of up to 5% for 7 days and did not show any toxic side effects like erythema on skin surface. Thus, the prepared extracts were considered safe for topical application on nude mice.

### 2.4. Balb/c Nude Mice

Athymic Balb/c male nude mice at 7 weeks of age were purchased from Dae-Han Biolink (Eumsung, Korea) and maintained under 12 h light: dark periods at 24 ± 2°C and were housed and handled under aseptic conditions. Experiment was performed in accordance with approved institutional protocols by the Institutional Animal Care and Use Committee (IACUC), Chungnam National University, Daejeon, South Korea. The authorization code number is CNU-00244.

### 2.5. Treatment Protocol of Different Natural Extracts on Nude Mice

Five animals were allocated to five randomized groups for evaluation of hair growth and hair changing pattern of nude mice. Topical application was performed daily with a solution of* A. radix, E. alba*, and* P. ginseng* extracts (2.5%) in a vehicle formulation and/or minoxidil (2%) until completion of two full hair growth cycles.

### 2.6. Measurement of Hair Density and Length

After daily treatment, in each hair growth cycle the images from specific regions of neck, back, and hip were captured two times per week using KONG, Bom-Viewer Plus (Seoul, Korea). About six images of 80× and 200× were exploited to measure the hair length and hair density, respectively, using Bom-Viewer Image Analyzer.

### 2.7. Evaluation of Hair Existing Area

The hair existing area of nude mice was systematically evaluated for each mouse three times per week. At this time point, mice were scored on a scale of “0” to “4” for percent body coverage of hair “0” = complete alopecia; “1” = <25% hair coverage; “2” = 25–50% hair coverage; “3” = 50–75% hair coverage; “4” = normal hair density and full hair coverage.

### 2.8. Histologic Assessment of Hair Growth

Mice were sacrificed in anagen phase and skin samples were fixed in 10% buffered formalin for histological analysis. Paraffin-embedded 4 *μ*m sections were stained with hematoxylin and eosin (H&E). The nude mice HF morphology and the number of HFs were evaluated microscopically in 5 fields per section of the dorsal skin at a magnification of ×100.

### 2.9. BrdU Immunohistochemistry

BrdU labeling cell proliferation was detected by intraperitoneal injection of bromodeoxyuridine (BrdU; Sigma, USA) with 50 mg/kg body weight at a concentration of 5 mg/mL in PBS. BrdU incorporation was detected by IHC staining of paraffin-embedded sections with mouse anti-BrdU primary antibody (Santa Cruz Biotechnology, Santa Cruz, CA, USA). Skin sections that were not incubated with primary antibodies were used as a negative control. Detection of follicular keratinocyte BrdU labeling was done at magnification ×100 and ×400.

### 2.10. Statistical Analysis

Statistical analysis was performed using SAS Systems ver. 9.3, and analysis of variance (ANOVA) was used for comparisons among all groups. All data are expressed as means ± SD unless otherwise is noted. A probability level of 5% was considered significant. Differences between two groups were analyzed by* t*-test. ANOVA was followed after Duncan's multiple comparison tests.

## 3. Results

### 3.1. Differences in Activity of Plant Extracts on Changing Hair Growth Pattern of Nude Mice

**Figure 1:**
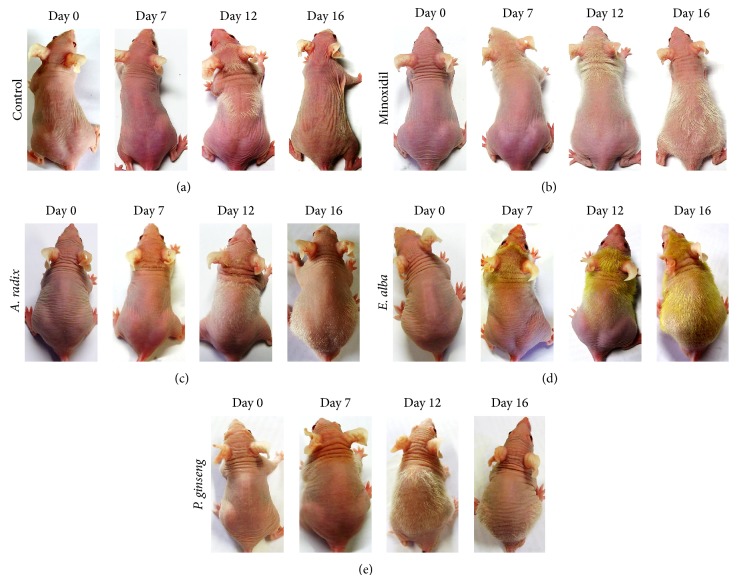
Stimulation of hair growth by different natural extract in nude mice. Specific concentrations of different extract in vehicle formulation were daily applied topically (*n* = 5) on the back of nude mice skin (a) control, (b) minoxidil, (c)* A. radix*, (d)* E. alba*, and (e)* P. ginseng* during one hair growth phase.

After daily treatment, hair growth was documented on experimental day 7. Systematic monitoring of hair growth showed that* A. radix* (Figure 1(c)) and vehicle treated control mice (Figure 1(a)) exhibit extremely sparse and transient hair coat. On the other hand, minoxidil and* E. alba* extract treated mice hair growth first documented from the frontal part of the head as early as 7 days and became dense, thicker, and longer that evenly extended to the shoulder and posterior part of the back during experimental day 12. The maximum hair coat was evident in* E. alba* extract treated group on experimental day 16 (Figures 1(d) and 1(b)). In contrast,* A. radix* (Figure 1(c)), and* P. ginseng* (Figure 1(e)) treated mice found rapid hair loss with increasing time point and eventually remained only few sparse, abortive short regional hair that moved caudally to the tail region.

### 3.2. Effect on Hair Coverage Area

**Figure 2:**
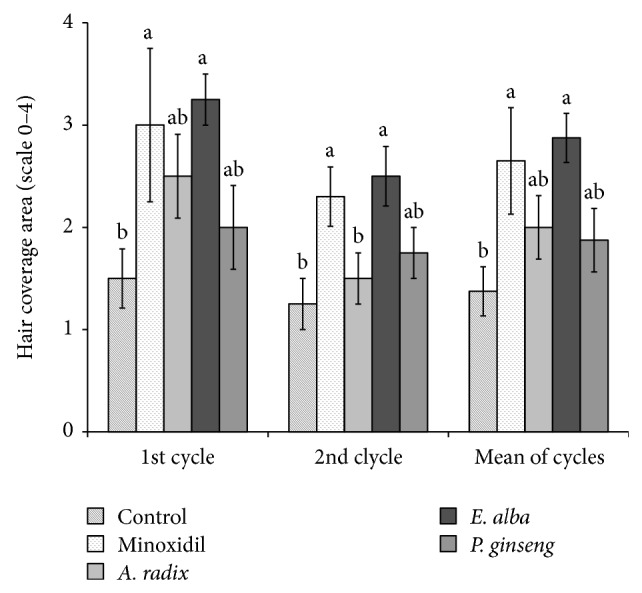
Effect of different plant extract on hair coverage area in athymic nude mice. Mice were evaluated on a scale of “0–4” parameters as follows: “0” = complete alopecia; “1” = <25% hair coverage; “2” = 25–50% hair coverage; “3” = 50–75% hair coverage; “4” = full hair coverage. The different letters (a and b) within a column indicate significant differences (*P* < 0.05) determined by Duncan's multiple range test.

The effects on hair coverage area of all the treated groups were precisely estimated for each mouse in both first and second hair growth cycle by giving them a score from “0” to “4.” The maximum hair growth score was significantly (*P* < 0.001) increased in mice treated with minoxidil and* E. alba* than control group during first and second hair growth cycles (Figure 2). On the other hand,* A. radix* treated group also consistently effective for inducing hair coverage area (2.25 ± 0.82) in first hair cycle but transient hair coat and progressive hair loss turn to similar score (1.25 ± 0.5) with that of control group (1.25 ± 0.5) in second hair cycle. There was no significant difference in terms of the hair coverage area among* P. ginseng* and control group.

### 3.3. Effect on Hair Length and Hair Density

**Figure 3:**
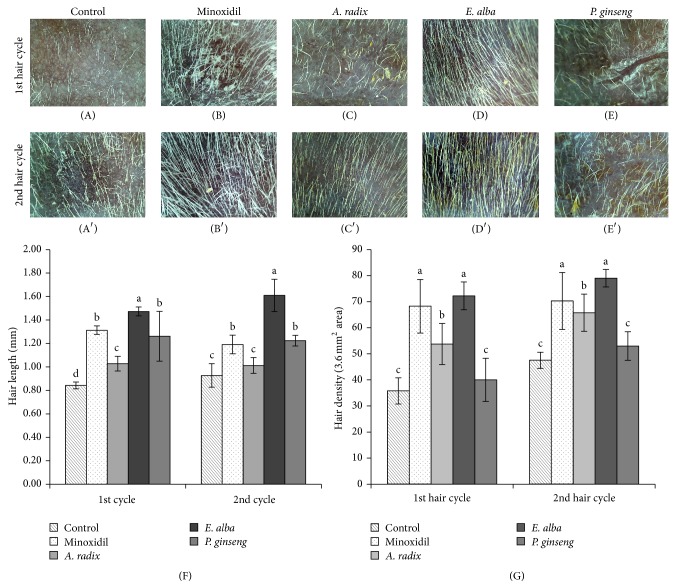
Evaluation of hair length and hair density in cycles. Image of nude mice hair on skin surface, treated with control ((A), (A′)), minoxidil ((B), (B′))*, A. radix* ((C), (C′))*, E. alba* ((D), (D′)), and* P. ginseng* ((E), (E′)) in first and second hair cycles. Hair length (F) and hair density (G) shown in column. The different letters (a, b, c, and d) within a column indicate significant differences (*P* < 0.0001) determined by Duncan's multiple range test. Digital images were takenby KONG, Bom-Viewer Plus with 80× magnification lens. Hair length and hair density were measured using Bom-Viewer Image analyzer (*n* = 10).

In terms of the measurement of hair length, the remarkable effect was observed in* E. alba* extract treated group. A significant increase (*P* < 0.0001) of hair length was observed in both first and second hair growth cycles while being compared with that of control group (Figure 3(F)). Similar effect was also found while evaluating hair density (Figure 3(G)). Although minoxidil treated group showed similar hair density with* E. alba* treated group (Figure 3(G)), the irregular and deformed hair was found in minoxidil treated mice (Figure 3(B)). On the other hand,* E. alba* treated mice hair (Figures 3(D) and 3(D′)) was straight and thicker and smother than other treatment groups. Although the hair length of* A. radix* and* P. ginseng* extract treated mice increased significantly than control group in earlier hair cycle, it became similar with that of control group afterward (Figure 3(F)). On the other hand, in terms of hair density* A. radix* treated group found significant effect compared to control but there was no difference of hair density of* P. ginseng* and control group (Figure 3(G)). Moreover, irregularly distributed single deformed hair shaft was also observed on the skin surface in both* A. radix* (Figures 3(C) and 3(C′)) and* P. ginseng* (Figures 3(E) and 3(E′)) treated mice similar to control group (Figures 3(A) and 3(A′)).

### 3.4. Effect of Plant Extracts on the Development and Morphogenesis of Nude Mice HF

**Figure 4:**
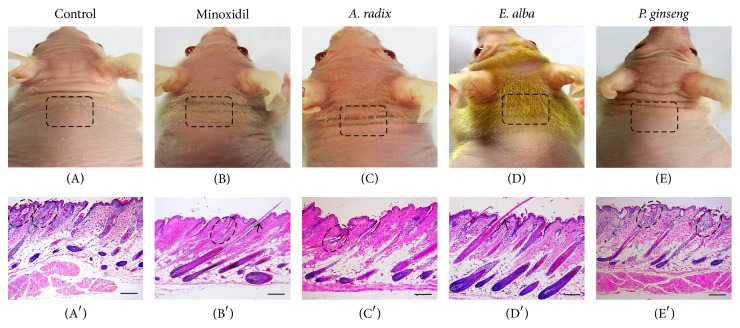
Histological features of the skin specimen of different extract and/or vehicle control treated mice. Macroscopic observation of hair in the skin surface of (A) control, (B) minoxidil, (C)* A. radix*, (D)* E. alba*, and (E)* P. ginseng* in early hair existing phase. Microscopic observation of longitudinal sections of (A′) control, (B′) minoxidil, (C′)* A. radix*, (D′)* E. alba,* and (E′)* P. ginseng* treated mice skin was harvested and fixed in buffered formalin for routine hematoxylin and eosin (H&E) stained histology (*n* = 7). The broken line indicates defective HFs at the level of sebaceous gland ((A′), (B′), (C′), and (E′)). Note that the straight hair shafts that emerge from the skin surface of minoxidil and* E. alba* treated mouse ((B), (D) and Arrows (B′), (D′)). Scale bar 100 *μ*m.

Histological examination of the skin specimen revealed differences between control and treated mice in the morphology of the developing HFs. On experimental day 10, minoxidil (Figure 4(B′)),* A. radix* (Figure 4(C′)), and* E. alba* treated mice (Figure 4(D′)) showed an early onset of anagen hair morphogenesis compared with that of control (Figure 4(A′)), confirming the macroscopic observation (Figures 4(B), 4(C), and 4(D)). On the other hand,* P. ginseng* extract treated mice skin specimen (Figure 4(E′)) showed similar stage of hair morphogenesis with that of control mice. Following the guidelines proposed by [19], we determined that the control and* P. ginseng* treated mice HFs were on stage 6 (Figures 4(A′) and 4(E′)) whereas* E. alba* extract treated mice HFs were clearly at stage 8 (Figure 4(D′)). Negligible numbers of hair shafts, which emerged from the HFs and moderately atrophic, were also observed in the skin of* A. radix*,* P. ginseng*, and vehicle treated mice (Figures 4(C), 4(E), and 4(A)). Moreover, the hair shafts of those were defective (often twisted) with dilation of the hair canal and apparently had insufficient structural rigidity to emerge through the epidermis (Figures 4(A′) and 4(E′)). On the other hand,* E. alba* treatedmice seemed to have a propensity for follicular accumulation of keratin and structural rigidity to penetrate the epidermis (Figure 4(D)).

### 3.5. Number of Hair Follicles

**Figure 5:**
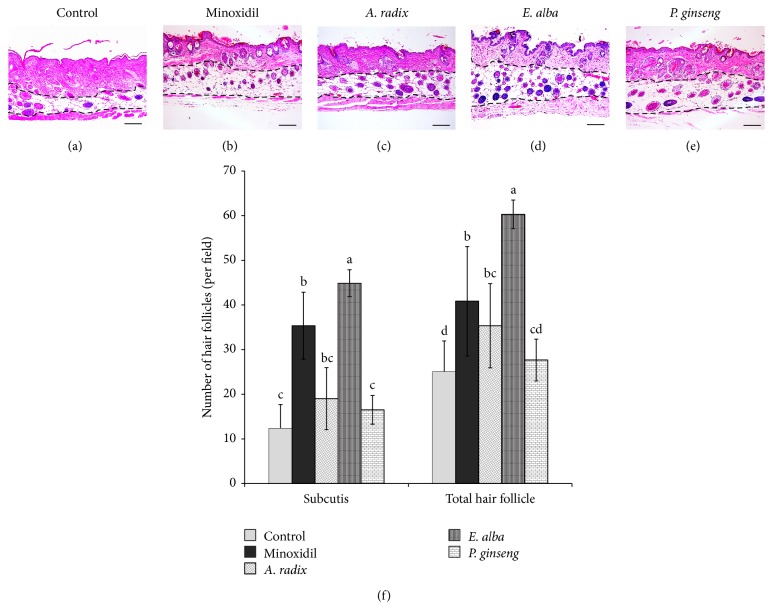
Increase in number of HFs in the dorsal skin of nude mice treated with different plant extract. Representative photomicrographs of the transverse sections of nude mice skin: (a) control, (b) minoxidil, (c)* A. radix*, (d)* E. alba*, and (e)* P. ginseng,* were stained with hematoxylin-eosin (H&E). (f) The number of hair follicles in subcutis with respect to the total number of HFs per field (*n* = 10). Values are mean ± standard deviation (S.D). The different letters (a, b, c, and d) within a column indicate significant differences (*P* < 0.0001) determined by Duncan's multiple range test. Scale bar 100 *μ*m.

Histologic assessment was performed to investigate the progression of hair follicles in nude mice, since there was an increase in the number of hair follicles observed in the deep subcutis during the anagen phase. In the representative transverse sections, the numbers of hair follicles were increased significantly in* E. alba* (*P* < 0.0001) and minoxidil (*P* < 0.005) extract treated groups compared to the control (Figure 5(f)).

### 3.6. BrdU Immunohistochemistry

**Figure 6:**
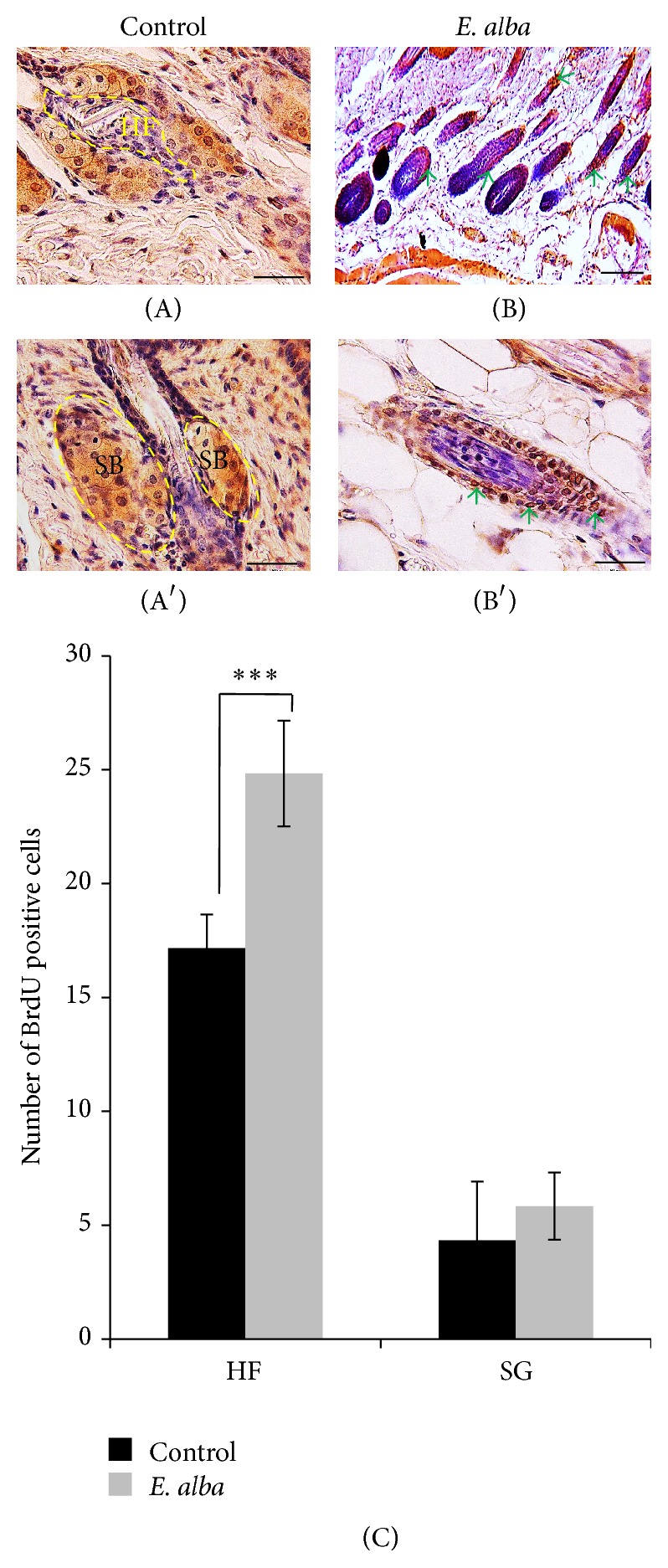
BrdU-labeled proliferating cells in anagen hair follicles. Control (A) and* E. alba* extract treated (B) follicles are nuclear BrdU staining (Arrows, red-brown). Scale bar, 100 *μ*m (B) and 50 *μ*m ((A), (A′), and (B′)). Number of BrdU positive cells (C). Data shown are means of five replicate samples (^***^ *P* < 0.001).

To further investigate the molecular mechanism underlying the ability of* E. alba* extract to form nude mice hair follicles, we performed immunohistochemistry with BrdU antibodies of the back skin of nude mice. We found that* E. alba* treated mice skin exhibits BrdU positive keratinocyte in the HFs, particularly a single cell layer that surrounds the innermost layer of the outer root sheath, as shown in Figures 6(B) and 6(B′). Moreover,* E. alba* treated nude mice exhibited a significant (*P* < 0.0001) increase in the number of BrdU-labeled keratinocyte per anagen follicle versus control mice (Figure 6(C)). On the other hand, there was no significant difference in the number of BrdU-labeled epithelial cells per sebaceous gland.

### 3.7. Identification and Quantification of Wedelolactone in* E. alba* Extract

**Figure 7:**
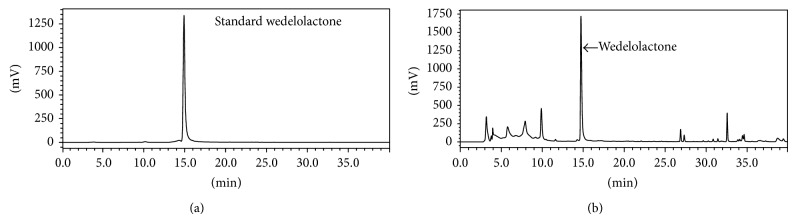
HPLC chromatogram. (a) Standard wedelolactone; (b) HPLC chromatogram of methanol extract of* E. alba.*

Additionally, HPLC analysis was performed to illustrate the methanol extract of* E. alba* (Figure 7) and was found to contain principle constituents like wedelolactone. The existence of the wedelolactone was quantified by 1.8% in methanol extract. It has been reported that various bioactive phytochemicals found in the* E. alba* extract, including wedelolactone, demethylwedelolactone, and saponins, are responsible for the main medicinal effects of* E. alba*, such as antitumor activity [13], antihepatitis C virus [12], and neuropharmacological activity [14] and its anticancer effect shows antiproliferative and cytotoxic effects determined using MTT assay [15].

## 4. Discussion

Medicinal plants may have potential by identifying active biological components and their successful use might open reverse pharmacology, a new approach to drug discovery. Given the known therapeutic properties of* E. alba* extract, we have performed enormous screening of different plant extracts on Balb/C nude mice model, and this has been used as a unique model to study the hair growth mechanism [20, 21].

Among all the plant extracts,* E. alba* showed outstanding hair growth promoting potential. Topical application was performed on the back of nude mice skin daily up to completion of two hair growth generations. The macroscopic appearance and the local distribution of hair were precisely observed in each individual group and showed clearly distinguishable feature. The regional distribution of the “abortive” anagen hair pattern in nude strains corresponded to the wave-like pattern of hair generations [22]. The hair growth area of* E. alba *treated mice is distinctly visible and simultaneously covered the maximum part of the back. In contrast,* A. radix, P. ginseng* treated mice with increasing age rapidly developed hair loss recognizable from the boundaries of hair covering. However, a few uneven hairs are visible particularly in nude mice skin surface, which referred to as “abortive hair growth” [23]. During anagen growth phase, we were able to observe a comparable growth pattern in* E. alba* treated mice with other treated and control mice (Figure 1). These phenomena may suggest that* E. alba* improved the disordered keratinization of nude mouse hair follicles, because original control hair shafts are short and irregular in shape suggesting some disordered keratinization of the HF [24, 25]. The precise genetic defect of nude mice, Foxn1 mutation expression, involved abnormal keratinization as the cause for defective cell type in the hair development [26–28]. Our observation suggests that treatment with* E. alba* acts transiently to normalize keratinocyte of nude mice HF, leading to fully formed visible HF with increased hair coverage area. An increase in the number and size of HFs has been considered as an indicator for the transition of hair growth from telogen to anagen [9, 19]. These results suggest that topical application of* E. alba* extract could stimulate the formation of hair follicles and induce an earlier anagen phase, compared to either the control or other treated groups (Figure 4). To elucidate the molecular mechanism underlying the ability of* E. alba* extract, the BrdU immunohistochemistry result suggests an enhanced keratinocyte proliferation in anagen hair follicles [29]. In our present studies, macroscopic analysis of hair growth parameters included changing pattern of nude mice hair growth, hair coverage area, and hair length. Histological study of different plant extracts along with control and minoxidil groups was investigated and found to have fully formed HFs in* E. alba* treated group as well as increasing the number of HFs. The active phase of HF cycling is also accompanied by increase in size and number of follicles resulting in an enlargement of the subcutis layer [19]. Given the vital role of hair restorer efficacy of* E. alba* extract, further HPLC analysis was carried out and showed that wedelolactone and other components exist in methanol extract. In this experiment, none of the plant extracts caused adverse dermatological effects, such as erythema, redness, drying or scaling, and dermatitis as it was case with minoxidil group at the skin site of nude mice.

It has been argued that combination of several constituents of a plant extract may act synergistically rather than single compound [30]. The interaction is possibly due to the binding of more than one compound at different sites on the same target or receptor. Further detailed clinical trials and studies will be necessary to investigate what components in* E. alba* extract contribute to its efficacy, since whole* E. alba* extract, rather than individual components, was used here to establish its biological activity against alopecia.

## 5. Conclusion

*E. alba* extract containing active ingredients provided more safety and compatibility which has more accessibility in pharmaceutical and cosmetic science and may promote the herbal medicine with minimal or no side effects or toxicities for hair growth therapy. Taking together all the investigation indicates that plant extract with suitable vehicle may be useful as an alternative topical medicine to minoxidil therapy.

## Figures and Tables

**Table 1:** Botanical origins, plant parts, and percentage yields of investigated specimens.

Botanical name (family)	Chinese name	Common name	Plant part	Extraction yield (% w/w)
*Eclipta alba* (Asteraceae)	Han lian cao	False daisy	Leaves, stems, and blooms	12.40
*Asiasarum sieboldii* (Aristolochiaceae)	Shashin	Chinese wild ginger root	Roots	7.52
*Panax ginseng* (Araliaceae)	Hong-Shen	Red ginseng	Roots	38.33
